# Quantitative and multiplexed DNA methylation analysis using long-read single-molecule real-time bisulfite sequencing (SMRT-BS)

**DOI:** 10.1186/s12864-015-1572-7

**Published:** 2015-05-06

**Authors:** Yao Yang, Robert Sebra, Benjamin S Pullman, Wanqiong Qiao, Inga Peter, Robert J Desnick, C Ronald Geyer, John F DeCoteau, Stuart A Scott

**Affiliations:** Department of Genetics and Genomic Sciences, Icahn School of Medicine at Mount Sinai, New York, NY 10029 USA; Icahn Institute for Genomics and Multiscale Biology, Icahn School of Medicine at Mount Sinai, New York, NY 10029 USA; Cancer Stem Cell Research Group, University of Saskatchewan, Saskatoon, SK S7N 4H4 Canada

**Keywords:** DNA methylation, CpG islands, Bisulfite sequencing, Long-read sequencing, Third generation sequencing, Single-molecule real-time (SMRT) sequencing, Pacific Bioscience

## Abstract

**Background:**

DNA methylation has essential roles in transcriptional regulation, imprinting, X chromosome inactivation and other cellular processes, and aberrant CpG methylation is directly involved in the pathogenesis of human imprinting disorders and many cancers. To address the need for a quantitative and highly multiplexed bisulfite sequencing method with long read lengths for targeted CpG methylation analysis, we developed single-molecule real-time bisulfite sequencing (SMRT-BS).

**Results:**

Optimized bisulfite conversion and PCR conditions enabled the amplification of DNA fragments up to ~1.5 kb, and subjecting overlapping 625–1491 bp amplicons to SMRT-BS indicated high reproducibility across all amplicon lengths (r = 0.972) and low standard deviations (≤0.10) between individual CpG sites sequenced in triplicate. Higher variability in CpG methylation quantitation was correlated with reduced sequencing depth, particularly for intermediately methylated regions. SMRT-BS was validated by orthogonal bisulfite-based microarray (r = 0.906; 42 CpG sites) and second generation sequencing (r = 0.933; 174 CpG sites); however, longer SMRT-BS amplicons (>1.0 kb) had reduced, but very acceptable, correlation with both orthogonal methods (r = 0.836-0.897 and r = 0.892-0.927, respectively) compared to amplicons less than ~1.0 kb (r = 0.940-0.951 and r = 0.948-0.963, respectively). Multiplexing utility was assessed by simultaneously subjecting four distinct CpG island amplicons (702–866 bp; 325 CpGs) and 30 hematological malignancy cell lines to SMRT-BS (average depth of 110X), which identified a spectrum of highly quantitative methylation levels across all interrogated CpG sites and cell lines.

**Conclusions:**

SMRT-BS is a novel, accurate and cost-effective targeted CpG methylation method that is amenable to a high degree of multiplexing with minimal clonal PCR artifacts. Increased sequencing depth is necessary when interrogating longer amplicons (>1.0 kb) and the previously reported bisulfite sequencing PCR bias towards unmethylated DNA should be considered when measuring intermediately methylated regions. Coupled with an optimized bisulfite PCR protocol, SMRT-BS is capable of interrogating ~1.5 kb amplicons, which theoretically can cover ~91% of CpG islands in the human genome.

**Electronic supplementary material:**

The online version of this article (doi:10.1186/s12864-015-1572-7) contains supplementary material, which is available to authorized users.

## Background

DNA methylation plays an essential role in several critical cellular processes including gene expression regulation, imprinting and X chromosome inactivation, and deregulation of the epigenetic machinery has been directly implicated in both Mendelian disorders and tumorigenesis [1-4]. In mammals, DNA methylation predominantly occurs at the 5-position of the cytosine pyrimidine ring within a CpG dinucleotide. Many techniques to detect CpG methylation have been developed that can be broadly classified by underlying chemistry, including bisulfite conversion, methylation sensitive restriction enzyme analyses, and methylated DNA immunoprecipitation [5-9]. Each has its own advantages and disadvantages; however, bisulfite conversion has been the most widely used sample preparation method for DNA methylation analysis. Common methylation detection techniques coupled with bisulfite conversion include PCR-based assays, microarrays, sequencing and other approaches, which differ in their capacities for single-nucleotide resolution, quantitation, and throughput.

Since the initial 1992 report on CpG methylation detection using bisulfite Sanger sequencing [10], the technique has been widely used for methylation discovery and as a diagnostic assay for detecting methylation abnormalities at imprinting control regions and the CpG islands of specific genes [11,12]. Importantly, the ongoing advances in sequencing technology have resulted in additional platforms available to execute bisulfite sequencing, including pyrosequencing [13,14] and next-generation sequencing [15-17]. Although laborious and low-throughput, bisulfite Sanger sequencing historically has been one of the most accurate and quantitative techniques to assess CpG methylation. Coupled with PCR amplicon cloning, bisulfite Sanger sequencing allows for allele-specific CpG methylation assessment; however, this is offset by a costly and time-consuming protocol and an inability to multiplex. Bisulfite pyrosequencing offers a faster, reproducible and quantitative analysis of DNA methylation, but is restricted to short read lengths (~150 bp) with limited capacity for multiplexing [18]. Although a serial pyrosequencing technique using multiple primers has been developed for extended read lengths, it is still constrained by short PCR amplicon lengths [14]. Additionally, it should be noted that all standard bisulfite conversion-based CpG methylation detection methods cannot discriminate 5-hydroxymethylcytosine from 5-methylcytosine. Consequently, oxidative bisulfite sequencing [19] and microarray [20] methods have recently been developed to distinguish these related nucleotides.

In contrast to targeted bisulfite sequencing techniques, next-generation bisulfite sequencing, including reduced representation bisulfite sequencing (RRBS) [21] and whole-genome bisulfite sequencing [22], can profile DNA methylation across an entire genome by quantitatively assessing the majority of CpG sites in a single experiment. This unparalleled resolution in methylation detection is transforming the field of epigenomics; however, adopting these genome-wide approaches requires significant bioinformatics expertise and may be prohibitively expensive for certain applications given their low throughput. An example of multiplexed and targeted bisulfite sequencing using 454 next-generation sequencing chemistry has been reported; however, the utility of this highly quantitative method was restricted to read lengths of only ~130 bp [15].

To address the need for a quantitative and highly multiplexed targeted bisulfite sequencing method with long read lengths, we developed a technique that combines bisulfite conversion with third-generation single-molecule real-time (SMRT) sequencing. Coupled with an optimized long-range bisulfite amplification protocol and empowered by the long read lengths of SMRT sequencing (up to ~20 kb) [23], multiplexed SMRT bisulfite sequencing (SMRT-BS) can accurately measure CpG methylation across ~1.5 kb regions without the need for PCR amplicon subcloning. As a cost-effective alternative to other targeted bisulfite sequencing techniques, SMRT-BS is an efficient and highly quantitative method for DNA methylation analysis.

## Results and discussion

### SMRT Bisulfite Sequencing (SMRT-BS) procedure

**Figure 1:**
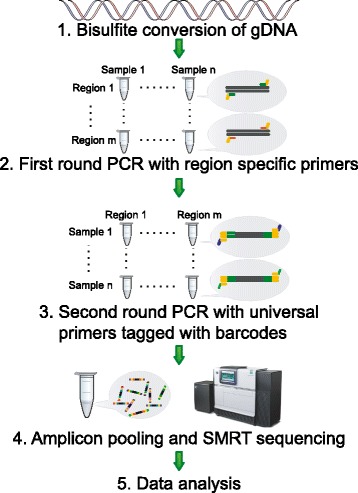
Illustration of the SMRT bisulfite sequencing (SMRT-BS) procedure. For detailed description of each step see Results and discussion.

The SMRT bisulfite sequencing (SMRT-BS) procedure consists of five steps (Figure 1): (1) bisulfite conversion of genomic DNA; (2) amplification of bisulfite-treated DNA using region-specific primers coupled with universal oligonucleotide tags; (3) re-amplification of amplicon templates using anti-tag universal primers coupled with sample-specific multiplexing barcodes; (4) amplicon purification, pooling and SMRT sequencing; and (5) CpG methylation quantitation.

### Bisulfite conversion

**Figure 2:**
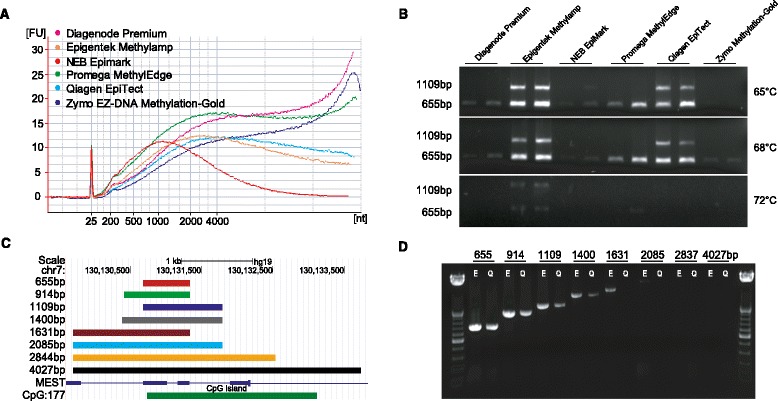
Bisulfite conversion and long-range amplification. **(A)** Six commercially available bisulfite conversion kits were tested and treated DNAs were examined using an Agilent 2100 Bioanalyzer to assess size distributions. **(B)** Two bisulfite-converted DNAs from each of the six kits were subjected to PCR with two amplicons (655 and 1109 bp) and three different extension temperatures (65°C, 68°C and 72°C) to assess capacity for long amplicon amplification. Bisulfite-converted DNA from two kits (Epigentek Methylamp and Qiagen EpiTect) with a PCR extension temperature of 65°C had the most robust amplification of the longer 1109 bp amplicon. **(C)** Eight amplicons ranging in size from 655–4027 bp (overlapping the *MEST* CpG island) were designed to determine the upper amplicon size limit of bisulfite PCR. **(D)** Agarose gel image of bisulfite PCR with the eight primer sets using DNA converted by the Epigentek Methylamp and Qiagen EpiTect kits revealing stable amplification of the 1631 bp amplicon with the Epigentek Methylamp converted DNA and the reported PCR conditions.

To maximize the capacity for long-read sequencing, sample preparation was optimized based on bisulfite conversion and PCR amplification. Six commercially available bisulfite conversion kits were tested and the size distributions of converted DNAs were assessed. Despite the DNA damage and fragmentation inflicted by bisulfite conversion [24], five out of the six bisulfite conversion kits resulted in DNA fragment distributions with peaks greater than ~2000 bp (Figure 2A), suggesting that longer amplicons than the typical bisulfite PCR size range (~300-500 bp) [25-27] may be achievable.

Since bisulfite conversion of DNA results in a substantial increase in adenine (A) and thymine (T) content and previous reports have indicated that A/T rich DNA can be more efficiently amplified by reduced PCR extension temperatures [28], long-range bisulfite PCR was evaluated using a gradient of extension temperatures. Treated DNA from each of the bisulfite conversion kits were subjected to PCR with two amplicon lengths (655 and 1109 bp) and extension temperatures of 65°C, 68°C, and 72°C (Figure 2B). These data indicated that two bisulfite conversion kits (Epigentek Methylamp and Qiagen EpiTect) and a PCR extension temperature of 65°C resulted in the most consistent amplification of the longer 1109 bp product using the reported conditions.

### Long-range amplification and SMRT-BS quality filtering

To determine the maximum PCR amplicon size achievable with bisulfite converted DNA from the Qiagen and Epigentek kits, eight primer sets were tested that ranged in amplicon length from 655–4027 bp (Figure 2C, Additional file 1: Table S1). All primers were previously validated by short-length PCR to ensure their capacity for amplification. These results indicated that bisulfite PCR with DNA converted by the Epigentek Methylamp kit using the reported conditions could amplify products up to ~2.0 kb (Figure 2D), although amplification of the 2.0 kb product was not generally consistent. The ~1.5 kb product was consistently amplified and was determined to be the approximated upper limit of stable bisulfite PCR using these conditions.

Notably, the ability to amplify longer amplicons following bisulfite conversion with the Epigentek Methylamp kit was not reflective of poor bisulfite conversion efficiency as the cytosine conversion rate of amplicons subjected to SMRT-BS (detailed below) had an average conversion rate of 97.0%. All amplicons with conversion rates less than 95% (6.8% of all reads) were filtered prior to CpG methylation quantitation, which increased the average conversion rate to 97.3%. In addition, potential clonal PCR artifacts, as defined by identical patterns of both CpG and non-CpG cytosines throughout the amplicons (0.3% of all reads) [29], were filtered prior to CpG methylation quantitation.

### Long-read SMRT-BS and reproducibility

**Figure 3:**
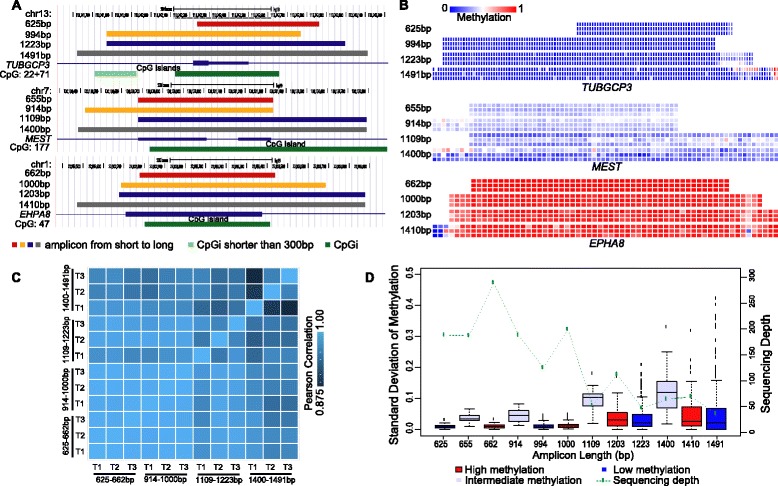
Long-read SMRT-BS and reproducibility. **(A)** To assess if amplicon length influenced methylation quantitation, CpG islands with previously determined low (*TUBGCP3*), intermediate (*MEST*), and high (*EHPA8*) methylation levels were subjected to SMRT-BS with four overlapping amplicons ranging from 625–1491 bp. Each amplicon was designed to completely or partially cover the CpG island (highlighted by green bars) and each was independently amplified in triplicate to determine reproducibility. **(B)** A heat map of CpG methylation identified by SMRT-BS for amplicons illustrated in **(A)**, demonstrating strong consistency with methylation quantitation between triplicate amplicons and between amplicon lengths. **(C)** A heat map of correlation between triplicate amplicons and across the different sized amplicons identifying strong overall correlation (0.972 ± 0.024), but reduced correlation with the longer amplicons. **(D)** Box plots of the standard deviation of CpG site methylation levels between triplicate amplicons for all tested regions (low methylation: blue; intermediate methylation: grey; high methylation: red). Of note, moderately increased variability in CpG site methylation levels was observed with the intermediately methylated CpG island (*MEST*) and with amplicons greater than ~1 kb (see Results and discussion). Overlaid is a green line graph of sequencing depth for each amplicon, suggesting preferential sequencing of the shorter amplicons compared to the longer amplicons and the influence of reduced sequencing depth on CpG site methylation level reproducibility.

To assess if long-range PCR amplification introduces any bias based on methylation level, CpG islands with low (*TUBGCP3*), intermediate (*MEST*), and high (*EHPA8*) methylation levels (previously determined by Illumina HumanMethylation450 BeadChip analysis) were subjected to SMRT-BS with four overlapping amplicons ranging from 625–1491 bp (Figure 3A; Additional file 1: Table S2). In addition, reproducibility was measured by simultaneously sequencing each of these four amplicons in triplicate using independent PCR reactions and unique barcodes. The methylation levels of all CpG sites interrogated were quantitated and are illustrated as a heat map in Figure 3B. The reproducibility of SMRT-BS was very high for all methylation levels and amplicon sizes, with an average overall correlation of r = 0.972 ± 0.024 between independent triplicate amplicons (Figure 3C). However, despite the overall high reproducibility, correlation with the longer amplicons was reduced compared to the correlation observed between shorter amplicons (Figure 3C).

In addition to assessing the correlation between overall amplicon methylation, individual CpG site methylation levels were compared and the average standard deviations (SDs) between triplicates in each tested amplicon are illustrated in Figure 3D. Of note, the median SDs for all CpG sites across all triplicate amplicons was less than ~0.10. The unmethylated and highly methylated CpG islands were very consistent at all amplicon lengths and replicates (median SD = 0.009, 0.009, 0.023 and 0.016 for the unmethylated amplicons, and SD = 0.010, 0.012, 0.032 and 0.024 for the highly methylated amplicons) (Figure 3D). However, analysis of the CpG island with an intermediate methylation level using the four different sized amplicons identified CpG site methylation median SDs of 0.044, 0.040, 0.097 and 0.111, respectively.

As also illustrated in Figure 3d, the amplicons that were greater than ~1 kb had lower SMRT-BS sequencing depth than the shorter amplicons despite being pooled with equal numbers of molecules. This resulted in methylation reproducibility being positively correlated with sequencing depth and negatively correlated with amplicon length. Consequently, these data underscore the need for increased sequencing depth for regions with intermediate methylation levels compared to those with low or high methylation levels to achieve similar confidence intervals and margins of error. Shorter amplicons may get preferentially sequenced when pooled with longer amplicons, which could be counterbalanced by increasing the molecule quantity of longer amplicons in the SMRT sequencing reaction when multiplexed with shorter amplicons. In addition, the increased variability observed in the intermediate methylation amplicons also suggests that PCR bias towards unmethylated DNA may occur, as has previously been reported with traditional bisulfite sequencing [30].

### Validation of SMRT-BS methylation quantitation

**Figure 4:**
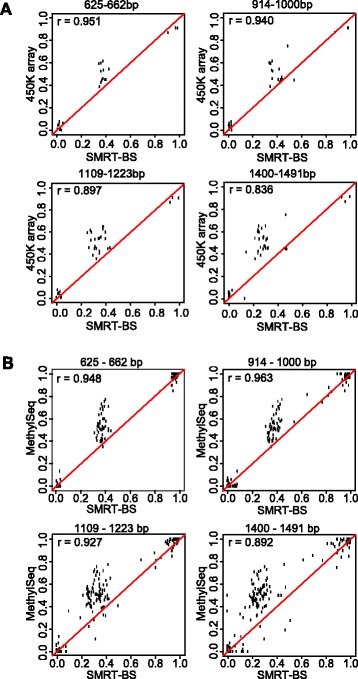
SMRT-BS validation. **(A)** Methylation quantitation by SMRT-BS of 42 CpG sites was compared to available data on the same sample using the HumanMethylation450 BeadChip (450K Array), resulting in an overall correlation of 0.906 ± 0.052. Correlation analyses were stratified by amplicon lengths, indicating a reduction in correlation with longer amplicons and possible PCR bias towards unmethylated DNA for the intermediate methylation region (see Results and discussion). **(B)** Methylation quantitation by SMRT-BS of 174 CpG sites was compared to available data on the same sample using the SureSelect™ Human Methyl-Seq target enrichment next generation sequencing platform (MethylSeq), resulting in an overall correlation of 0.933 ± 0.031. Correlation analyses were stratified by amplicon lengths, indicating a reduction in correlation with longer amplicons and possible PCR bias towards unmethylated DNA for the intermediate methylation region (see Results and discussion).

Validation of SMRT-BS was accomplished by comparing identified *TUBGCP3*, *MEST*, and *EHPA8* CpG methylation levels from peripheral blood DNA samples with methylation data on the same samples from two independent technologies, the HumanMethylation450 BeadChip (42 CpG sites) and the SureSelect™ Human Methyl-Seq target enrichment next-generation sequencing platform (174 CpG sites). The SMRT-BS methylation levels of these CpG sites ranged from 0 to 1.0 and had an average sequencing depth of 106X. Correlation analyses indicated that the identified SMRT-BS methylation levels were consistent with the two orthogonal technologies with an overall r = 0.906 ± 0.052 and r = 0.933 ± 0.031, respectively. The lower correlation observed with the HumanMethylation450 BeadChip data was likely due to fewer CpG sites included in the analysis. Stratifying the correlation analyses by amplicon length indicated that although the overall correlations were very good for all lengths, reduced correlation was observed with the amplicons greater than 1.0 kb than those less than 1.0 kb (Figure 4). These results are consistent with the increased SD observed between replicates of the longer amplicons (Figure 3D) and are likely influenced, in part, by lower sequencing depth compared to the shorter amplicons (as noted above). However, the pattern of shifted intermediate methylation levels observed in the validation analyses also support the aforementioned possibility of PCR bias towards unmethylated DNA that has been previously reported with amplicon bisulfite sequencing [30].

### SMRT-BS multiplexing and utility

**Figure 5:**
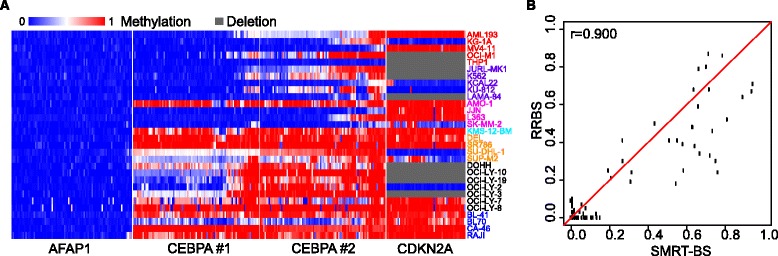
SMRT-BS utility and multiplexing. **(A)** Heat map of SMRT-BS methylation quantitation results for four CpG island amplicons [*AFAP1* (798 bp), *CEBPA* (702 bp and 866 bp), *CDKN2A* (770 bp)] and 30 hematological malignancy cell lines. *CDKN2A* deletion is illustrated by grey bars. **(B)** SMRT-BS methylation results of 79 CpG sites for the K562 cell line were compared to available RRBS data from the ENCODE project [31], revealing a correlation of 0.900.

To evaluate the utility of SMRT-BS with multiplexed amplicons and specimens, four distinct CpG island amplicons [*AFAP1* (798 bp), *CEBPA* (702 bp and 866 bp), *CDKN2A* (770 bp)] and 30 hematological malignancy cell lines were simultaneously subjected to SMRT-BS (Additional file 1: Table S3). The average sequencing depth was 110X for each of the 120 amplicons, which identified a spectrum of highly quantitative methylation levels across all cell lines and interrogated CpG sites (Figure 5A). The *CDKN2A* tumor suppressor gene is commonly deleted in hematological cancers and those cell lines with *CDKN2A* deletions could not amplify products. Of note, one of the cell lines included in the SMRT-BS multiplexing analysis (K562) was previously subjected to RRBS methylation analysis through the publically available ENCODE project [31]. There were 129 CpG sites covered in this cell line by both data sets, with average SMRT-BS and RRBS sequencing depths of 101X and 31X, respectively. Among these CpG sites, 78 had >20X coverage by RRBS, and the Pearson correlation between SMRT-BS and RRBS methylation levels among these 78 CpG sites was 0.900 (Figure 5B), further supporting the overall accuracy of SMRT-BS methylation quantitation.

## Conclusions

Many techniques have been developed to measure CpG methylation, which differ widely in their chemistry, sample requirement, resolution, capacity for quantitation, throughput, accessibility and cost (for recent review, see [8]). Although genome-wide sequencing approaches are often desirable, the immediate benefits in CpG coverage can be offset by high per sample costs, a low throughput, and the need for computational expertise. Consequently, targeted approaches to detect CpG methylation are still widely used and continually developed, now often coupling bisulfite conversion with next-generation sequencing [15-17]. However, the paucity of targeted bisulfite sequencing techniques capable of examining amplicons longer than the typical bisulfite PCR size range (~300-500 bp) [15-17,25-27] prompted our development of SMRT-BS.

Although SMRT sequencing is capable of directly detecting modified nucleotides by their unique polymerase kinetics without bisulfite conversion [32], the introduction of an amplification step abolishes its ability to detect any epigenetic modifications of template DNA. A key component to the development of SMRT-BS was the optimization of bisulfite conversion and PCR, which resulted in amplicons up to ~1.5-2.0 kb from bisulfite-converted DNA. Subjecting these amplicons to multiplexed SMRT-BS indicated that the technique was reproducible and highly concordant with other lower throughput quantitative CpG methylation methods. However, the increased variability observed with SMRT-BS of intermediately methylated regions must be acknowledged as a potential limitation that may be mitigated by increased sequencing depth. Similarly, as has previously been reported for amplicon bisulfite sequencing [30], it should be noted that SMRT-BS of intermediately methylated regions may also be influenced by PCR bias towards unmethylated DNA. To account for this known problem inherent to amplicon bisulfite sequencing, calibration strategies using standard curves of methylation controls have been reported if needed [30,33].

As a targeted amplicon sequencing method and an extension of previous bisulfite sequencing techniques, SMRT-BS is capable of a high degree of multiplexing and sequencing depth for accurate methylation quantitation with negligable clonal PCR artifacts. Importantly, this technique also takes advantage of the long read lengths achievable with SMRT sequencing, which allows for more thorough regional CpG methylation assessment and increases the capacity for studying the relationship between phased single nucleotide variants and allele-specific CpG methylation. Additional potential applications of SMRT-BS include targeted panels for diagnostic CpG methylation detection, and multiplexed interrogation of candidate CpG methylation sites for epigenome-wide association study (EWAS) [34,35] replication analyses. Notably, the size distribution of all CpG islands in the hg19 human genome reference sequence (as defined by the UCSC Genome Browser and reference [36]) suggests that the ability of SMRT-BS to measure DNA methylation across ~1.5 kb amplicons could theoretically interrogate ~91% of all the CpG islands in the human genome.

## Methods

### Specimens and cell lines

Peripheral blood samples from healthy donors who self-reported their racial background and gave informed consent for the use of their DNA for research were obtained from the New York Blood Center with IRB approval as previously defined [37]. All personal identifiers were removed, and isolated DNA samples were tested anonymously. Genomic DNA was isolated using the Puregene® DNA Purification kit (Qiagen, Valencia, CA) according to the manufacturer’s instructions.

The human acute myeloid leukemia (AML193, THP-1, KG1A, MV4-11, KCL-22, OCI-M1), chronic myeloid leukemia (LAMA-84, JURL-MK1, K562, KU-812), anaplastic large cell lymphoma (SU-DHL-1, SUP-M2, SR786, DEL), plasma cell leukemia (SK-MM-2, L-363, AMO-1, JJN), Burkitts lymphoma (BL-41, BL-70, CA-46, RAJI), B–cell lymphoma (DOHH, OCI-LY-2, OCI-LY-3, OCI-LY-7, OCI-LY-8, OCI-LY-10, OCI-LY-19) and multiple myeloma (KMS-12-BM) cell lines were obtained from either the Leibniz Institute DSMZ-German Collection of Microorganisms and Cell Cultures (Braunschweig, Germany) or the American Type Culture Collection (ATCC; Manassas, VA, USA). All cell lines were cultured as per recommended conditions. Genomic DNAs were isolated using the DNeasy Blood and Tissue Kit (Qiagen, Valencia, CA, USA) according to the manufacturer’s instructions.

### Bisulfite treatment

All bisulfite treatments of genomic DNAs (1 μg) were performed according to the manufacturer’s instructions. Six commercial kits were evaluated: (1) Diagenode Premium Bisulfite Kit, (2) Epigentek Methylamp DNA Modification Kit, (3) NEB EpiMark Bisulfite Conversion Kit, (4) Promega MethylEdge Bisulfite Conversion System, (5) Qiagen EpiTect Bisulfite Kit, and (6) Zymo EZ-DNA Methylation-Gold Kit. Bisulfite-treated DNAs were quantified using the NanoDrop 1000 and sized with a 2100 Bioanalyzer using the RNA6000 Pico kit (Agilent Technologies).

### Bisulfite PCR

First-step PCR reactions were performed in 20 μl containing ~50-100 ng of bisulfite-treated DNA, 1X PCR Buffer, 0.2 mM of each dNTP, 0.2 μM forward and reverse region-specific primers (Additional file 1: Tables S1-S3), and 1.0 unit of TaKaRa Taq HS (Clontech Laboratories, Inc., Mountain View, CA, USA). Amplification consisted of an initial denaturation step at 94°C for 2 min followed by 35 amplification cycles (94°C for 20 sec, 55°C for 45 sec, and 65°C for 1 min/kb + 30 seconds) and a final incubation at 65°C for 5 min (unless otherwise noted). Second-step PCR reactions were carried out using 1 μl of a 1:50 dilution of first-step PCR product in a total volume of 25 μl with the same conditions as first-step PCR, but using barcoded universal primers and an annealing temperature of 60°C.

### Single-Molecule Real-Time (SMRT) sequencing

All PCR amplicons were purified using the QiaQuick PCR Purification Kit (Qiagen) and quantified by Nanodrop 1000. After purification, PCR amplicons were pooled with equal amounts of molecule quantity. The required volume of each amplicon was calculated by the following formula:

$$ {V}_i=\frac{M\times {L}_i}{n\times {C}_i\times {\displaystyle {\sum}_{i=1}^m{L}_i}} $$

Where *V*_*i*_ is the volume of each PCR amplicon, *M* is the total mass of pooled PCR amplicons, *L*_*i*_ is the length of each amplicon, *n* is the total number of samples, *C*_*i*_ is the concentration of each amplicon and *m* is the total number of amplicons. A total of 500 ng of pooled PCR amplicons were submitted for SMRT sequencing.

Single-molecule real-time (SMRT) sequencing was performed according to the P5-C3 Pacific Biosciences protocol with a movie collection time of 180 minutes. In brief, pooled PCR amplicons were quantified using Qubit fluorometric analysis (Life Technologies) and a Bioanalysis 12000 chip (Agilent Technologies) to assess PCR amplicon quality, size, and quantity. Additionally, barcoded and pooled amplicons were purified using Ampure XP Solid Phase Reversible Immobilization (Beckman Coulter) at 0.8-fold volume. SMRTbell libraries were constructed using end-repair, ligation, and exonuclease purification strategies detailed in the Pacific Biosciences P5-C3 Template Preparation Kit protocols. SMRTbell templates were then bound to polymerase molecules for 4 hours at 25°C using 3 nM of the amplicon SMRTbell library and excess P5 DNA polymerase at a concentration of 9 nM as previously described [38]. The polymerase-template complexes were immobilized at 250 pM for 30 min on nanofabricated SMRTcells containing an array of zero-mode waveguides (ZMWs), and ZMWs were analyzed for sequencing to generate reads using a 1x180-minute collection protocol. Circular consensus sequencing (CCS) was employed using multiple passes on each SMRTbell to generate CCS reads with higher accuracy for data analysis. The ‘Reads of Insert’ pipeline was utilized with a filter of 85% accuracy and 1-pass prior to downstream analyses.

### SMRT-BS data analysis

**Figure 6:**
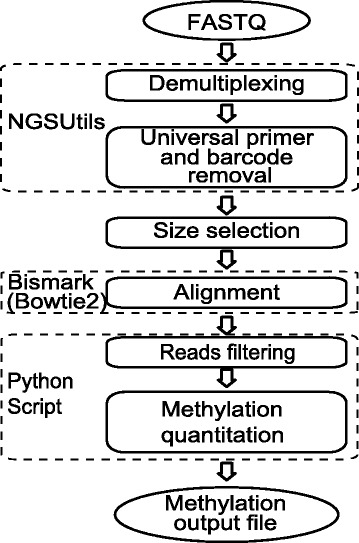
Illustration of the SMRT-BS analysis pipeline. For detailed description of each step see Methods.

The SMRT-BS data analysis workflow is illustrated in Figure 6. Briefly, sequencing reads in FASTQ format were demultiplexed and trimmed using NGSutils [39]. Two mismatches (including insertions/deletions) were allowed in each 18 bp barcode, and both universal primers and barcodes were trimmed prior to downstream analyses. Only reads longer than 50 bp were used for subsequent methylation extraction. Bismark [40] (with bowtie 2 [41]) was used for alignment, and the generated SAM files were subjected to read filtering (see Results and discussion) and CpG methylation quantitation using an in-house developed Python script. Any CpG sites with sequencing depth lower than 10X were excluded from further analyses. Please note that a freely available program to analyze SMRT-BS and other high-throughput bisulfite sequencing data (HiTMAP: High Throughput Methylation Analysis Program) is in development and a manuscript detailing its functionality and accessibility is in preparation.

### Infinium HumanMethylation450 BeadChip (450K-array)

Methylation data from SMRT-BS was validated against overlapping CpG site methylation data derived from the Infinium HumanMethylation450 BeadChip (450K-array; Illumina, Inc., San Diego, CA, USA). The samples were processed according to the manufacturer’s instructions by the Genomics Core Laboratory at the Icahn School of Medicine at Mount Sinai without any modification to the manufacturer’s protocol. 450K-array data were obtained and normalized using GenomeStudio® software (Illumina) according to the manufacturer’s instructions and the Illumina Methylation Analyzer (IMA) R package [42]. 450K-array probes with detection p-values greater than 0.01 or with missing values were removed prior to further analyses. Multiple-mapped probes with less than two mismatches or indels were identified by the Bowtie aligner and removed to avoid potential errors of unspecific binding. In addition, probes that included known single-nucleotide polymorphisms according to 450K-array annotation were removed and peak-based normalization was performed using IMA.

### SureSelect™ human Methyl-Seq

Methylation data from SMRT-BS was also validated against overlapping CpG site methylation data derived from the SureSelect™ Human Methyl-Seq (Agilent Technologies, Santa Clara, CA, USA) target enrichment system. Libraries were prepared according to the manufacturer’s instructions without any modification to the manufacturer’s protocol. In brief, 2 μg of genomic DNAs were sheared by a Covaris E210 to fragments of ~150 bp, end-repaired, 3’ end-adenylated and ligated with methylated adaptors. Pre-prepared DNA fragments were hybridized to SureSelect™ Human Methyl-Seq capture libraries, and target captured DNAs were treated with bisulfite, subjected to eight PCR cycles to enrich adaptor added fragments and six PCR cycles to add multiplexing barcodes. Sequencing was accomplished using a HiSeq 2000 (Illumina) and 100 bp single-read sequencing with one lane per two samples. Raw sequencing data was analyzed with Bismark [40] to extract DNA methylation information.

### Availability of supporting data

Data sets supporting the results of this study were uploaded to public data repositories for open access. The SMRT-BS sequencing FASTQ data are available from the NCBI Sequence Read Archive (SRA) using the SRX977650 and SRX977540 experiment identifiers: *http://www.ncbi.nlm.nih.gov/sra/SRX977650[accn]* and *http://www.ncbi.nlm.nih.gov/sra/SRX977540[accn]*. The 450K-array and Methyl-Seq validation data are available from the LabArchives Electronic Laboratory Notebook (DOI: 10.6070/H45H7D8C).

## Additional file

Additional file 1:
**Includes three Supplemental Tables listing the oligonucleotide primers used for bisulfite PCR.**

## Abbreviations

CCS: Circular consensus sequencing
RRBS: Reduced representation bisulfite sequencing
SMRT: Single-molecule real-time
SMRT-BS: SMRT bisulfite sequencing
SD: Standard deviation
ZMWs: zero-mode waveguides
