# Supplementary material for: Quantitative and multiplexed DNA methylation analysis using long-read single-molecule real-time bisulfite sequencing (SMRT-BS)
Source: BMC Genomics. 2015 May 6;16(1):350. doi: 10.1186/s12864-015-1572-7 (PMC4422326; doi:10.1186/s12864-015-1572-7)
Supplement: Additional file 1: — Includes three Supplemental Tables listing the oligonucleotide primers used for bisulfite PCR. [file 12864_2015_1572_MOESM1_ESM.docx]

**Quantitative and multiplexed DNA methylation analysis using long-read single-molecule real-time bisulfite sequencing (SMRT-BS)**

Yao Yang^1*^, Robert Sebra^1,2^, Benjamin Pullman^1^, Wanqiong Qiao^1^, Inga Peter^1^, Robert J. Desnick^1^, C. Ronald Geyer^2^, John F. DeCoteau^2^, and Stuart A. Scott^1*^

^1^ Department of Genetics and Genomic Sciences, Icahn School of Medicine at Mount Sinai, New York, NY 10029, USA.

^2^ Icahn Institute for Genomics and Multiscale Biology, Icahn School of Medicine at Mount Sinai, New York, NY 10029, USA.

^3^ Cancer Stem Cell Research Group, University of Saskatchewan, Saskatoon, SK, S7N 4H4, Canada.

**Correspondence to:**

**Yao Yang, PhD Stuart A. Scott, PhD**

E-mail: yao.yang@mssm.edu E-mail: stuart.scott@mssm.edu

**ADDITIONAL DATA FILES:**

**Supplemental Table S1:** Oligonucleotide primers used for bisulfite PCR amplification to assess upper limit of amplicon length

**Supplemental Table S2:** Oligonucleotide primers used for bisulfite PCR amplification to assess reproducibility and accuracy

**Supplemental Table S3:** Oligonucleotide primers used for bisulfite PCR amplification in hematological malignancy cell lines

**Supplemental Table S1: Oligonucleotide primers used for bisulfite PCR amplification to assess upper limit of amplicon length**

| **Gene** | **Sequence^*^** | **Region [hg19]** | **Length (bp)** | **CpG Sites** |
| --- | --- | --- | --- | --- |
| ***MEST*** | TTTGTGTTGTGTTAGAGGTTTTGAT | chr7:130130682-130131336 | 655 | 46 |
|  | TAAACCCACCACCAAACTAATAAAC |  |  |  |
|  | GGTTTTGTTTTTGAGGGTTTTATA | chr7:130130423-130131336 | 914 | 54 |
|  | TAAACCCACCACCAAACTAATAAAC |  |  |  |
|  | TTTGTGTTGTGTTAGAGGTTTTGAT | chr7:130130682-130131790 | 1109 | 68 |
|  | CCACAAAAATAAAATACCCCTCTAAC |  |  |  |
|  | GTTTTGGGGTTATAAAAGGTGAATAA | chr7:130130391-130131790 | 1400 | 76 |
|  | TAAACCCACCACCAAACTAATAAAC |  |  |  |
|  | ATGTGGGTAGATATGTTTTATGGTT | chr7:130129706-130131336 | 1631 | 69 |
|  | TAAACCCACCACCAAACTAATAAAC |  |  |  |
|  | ATGTGGGTAGATATGTTTTATGGTT | chr7:130129706-130131790 | 2085 | 91 |
|  | TAAACCCACCACCAAACTAATAAAC |  |  |  |
|  | ATGTGGGTAGATATGTTTTATGGTT | chr7:130129706-130132532 | 2827 | 165 |
|  | AATACCAAAATCTAAAAATCCCAATT |  |  |  |
|  | ATGTGGGTAGATATGTTTTATGGTT | chr7:130129706-130133732 | 4027 | 221 |
|  | TCCCAATATCTCCTTAAAAAATCAA |  |  |  |

* All primers have a universal tag at their 5’ end to enable the addition of barcodes through a second round PCR. The sequences for the universal forward and reverse primers are ATGGGTTCCAGAGTCAATCT and GAAAGGTCTGGAGTCTTGAT, respectively.

**Supplemental Table S2: Oligonucleotide primers used for bisulfite PCR amplification to assess reproducibility and accuracy**

| **Gene** | **Sequence^*^** | **Region [hg19]** | **Length (bp)** | **CpG Sites** |
| --- | --- | --- | --- | --- |
| ***EPHA8*** | TGAGTTTTAATTAGAATAATTGGTTG | chr1:22902777-22903438 | 662 | 47 |
|  | TAACAACCCTACCAAAACCAAAC |  |  |  |
|  | TGGTTGTGTTTTTTTTGTTTATAGTG | chr1:22902686-22903685 | 1000 | 57 |
|  | AAAATCCCATAACATACCCAATTAC |  |  |  |
|  | TTGTTGTAGTGGTTGTGTTTTTTTT | chr1:22902677-22903879 | 1203 | 60 |
|  | ACTCTACCATCCCCAACTACATAAC |  |  |  |
|  | TGGTTTATAGGTTAGAGTTTTTATTTTT | chr1:22902470-22903879 | 1410 | 63 |
|  | ACTCTACCATCCCCAACTACATAAC |  |  |  |
| ***TUBGCP3*** | TTTGTAGTATAGGTTTTGTAGTAGAA | chr13:113242238-113242862 | 625 | 62 |
|  | AACAAATAAACTACCCACTACAC |  |  |  |
|  | TTTTTATTTTATAGGATGAATTTAAAGG | chr13:113241774-113242767 | 994 | 108 |
|  | CATCAAAAATATAAATCAAACCAATACC |  |  |  |
|  | TTTTTATTTTATAGGATGAATTTAAAGG | chr13:113241774-113242996 | 1223 | 123 |
|  | ACAAATTTCCTATTCTCTCACTCC |  |  |  |
|  | TTTTTAATTTTTTAAATAGTAGGAAAATA | chr13:113241622-113243112 | 1491 | 137 |
|  | AAATTAACCTTTTAACATAATAACTCAC |  |  |  |
| ***MEST*** | TTTGTGTTGTGTTAGAGGTTTTGAT | chr7:130130682-130131336 | 655 | 46 |
|  | TAAACCCACCACCAAACTAATAAAC |  |  |  |
|  | GGTTTTGTTTTTGAGGGTTTTATA | chr7:130130423-130131336 | 914 | 54 |
|  | TAAACCCACCACCAAACTAATAAAC |  |  |  |
|  | TTTGTGTTGTGTTAGAGGTTTTGAT | chr7:130130682-130131790 | 1109 | 68 |
|  | CCACAAAAATAAAATACCCCTCTAAC |  |  |  |
|  | GTTTTGGGGTTATAAAAGGTGAATAA | chr7:130130391-130131790 | 1400 | 76 |
|  | CCACAAAAATAAAATACCCCTCTAAC |  |  |  |

* All primers have a universal tag at their 5’ end to enable the addition of barcodes through a second round PCR. The sequences for the universal forward and reverse primers are ATGGGTTCCAGAGTCAATCT and GAAAGGTCTGGAGTCTTGAT, respectively.

**Supplemental Table S3: Oligonucleotide primers used for bisulfite PCR amplification in hematological malignancy cell lines**

| **Gene** | **Sequence^*^** | **Region [hg19]** | **Length (bp)** | **CpG Sites** |
| --- | --- | --- | --- | --- |
| ***APAF1*** | TTGTTTTATTGAGTTTTTTAGTTGTTAGTT | chr12:99038641-99039438 | 798 | 87 |
|  | CCTCCCCTAAATCTCTACAACC |  |  |  |
| ***CEBPA*** | GGGGTAGTTTGGAGATTAGAGTTAG | chr19:33793316-33794017 | 702 | 91 |
|  | TCCATAAAAAAATTAAAATTCTCCC |  |  |  |
|  | GTTTTGTTAGGTTTAAGGTTATTGT | chr19:33794001-33794866 | 866 | 90 |
|  | AATCTCCAAACTACCCCTATAATTC |  |  |  |
| ***CDKN2A*** | GGTGGGGTTTTTATAATTAGGAAAG | chr9:21974658-21975427 | 770 | 57 |
|  | CTACAAACCCTCTACCCACCTAAA |  |  |  |

* All primers have a universal tag at their 5’ end to enable the addition of barcodes through a second round PCR. The sequences for the universal forward and reverse primers are ATGGGTTCCAGAGTCAATCT and GAAAGGTCTGGAGTCTTGAT, respectively.
